# Pinnisterols A–C, New 9,11-Secosterols from a Gorgonian *Pinnigorgia* sp.

**DOI:** 10.3390/md14010012

**Published:** 2016-01-07

**Authors:** Yu-Chia Chang, Liang-Mou Kuo, Tsong-Long Hwang, Jessica Yeh, Zhi-Hong Wen, Lee-Shing Fang, Yang-Chang Wu, Chan-Shing Lin, Jyh-Horng Sheu, Ping-Jyun Sung

**Affiliations:** 1 Doctoral Degree Program in Marine Biotechnology, National Sun Yat-sen University and Academia Sinica, Kaohsiung 804, Taiwan; jay0404@gmail.com (Y.-C.C.); wzh@mail.nsysu.edu.tw (Z.-H.W.); 2 National Museum of Marine Biology & Aquarium, Pingtung 944, Taiwan; 3 Graduate Institute of Clinical Medical Sciences, College of Medicine, Chang Gung University, Taoyuan 333, Taiwan; kuo33410@yahoo.com.tw; 4 Division of General Surgery, Department of Surgery, Chang Gung Memorial Hospital, Chiayi 613, Taiwan; 5 Graduate Institute of Natural Products, School of Traditional Chinese Medicine, College of Medicine and Chinese Herbal Medicine Research Team, Healthy Aging Research Center, Chang Gung University, Taoyuan 333, Taiwan; htl@mail.cgu.edu.tw; 6 Research Center for Industry of Human Ecology and Graduate Institute of Health Industry Technology, Chang Gung University of Science and Technology, Taoyuan 333, Taiwan; 7 Department of Marine Biotechnology and Resources, Asia-Pacific Ocean Research Center, National Sun Yat-sen University, Kaohsiung 804, Taiwan; jessicayeh8912001@gmail.com; 8 Department of Sport, Health, and Leisure, Cheng Shiu University, Kaohsiung 833, Taiwan; lsfang@csu.edu.tw; 9 School of Pharmacy, College of Pharmacy, China Medical University, Taichung 404, Taiwan; yachwu@mail.cmu.edu.tw; 10 Chinese Medicine Research and Development Center, China Medical University Hospital, Taichung 404, Taiwan; 11 Center for Molecular Medicine, China Medical University Hospital, Taichung 404, Taiwan; 12 Graduate Institute of Natural Products, Kaohsiung Medical University, Kaohsiung 807, Taiwan; 13 Graduate Institute of Marine Biology, National Dong Hwa University, Pingtung 944, Taiwan

**Keywords:** secosterol, gorgonian, *Pinnigorgia*, anti-inflammatory, superoxide anion, elastase, cytotoxicity, HSCs

## Abstract

Three new 9,11-secosterols, pinnisterols A–C (**1**–**3**), were isolated from a gorgonian coral *Pinnigorgia* sp., collected off the waters of Taiwan. The structures of these compounds were elucidated on the basis of spectroscopic methods. The new sterols **1** and **3** displayed significant inhibitory effects on the generation of superoxide anions and the release of elastase by human neutrophils, and sterol **1** was found to show moderate cytotoxicity in hepatic stellate cells (HSCs).

## 1. Introduction

**Figure 1:**
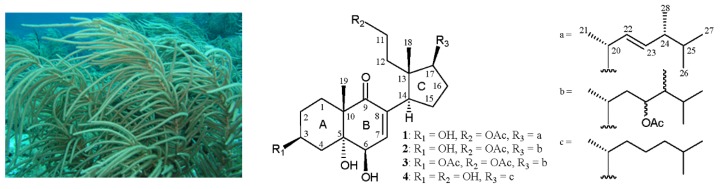
Gorgonian coral *Pinnigorgia* sp. and the structures of 9,11-secosterols **1**–**4**.

Studies on the chemical constituents of octocorals collected off the waters of Taiwan, at the intersection of the Kuroshio current and the South China Sea surface current, have led to the isolation of a series of interesting 9,11-secosterols from *Cespitularia hypotentaculata* [1], *Cladiella hirsuta* [2], *Sinularia granosa* [3], *Sinularia leptoclados* [4,5], *Sinularia lochmodes* [4], and *Sinularia nanolobata* [6]. Steroids of this type were found to possess interesting bioactivities, such as cytotoxic [1,2,3,4,5,6], anti-inflammatory [3] and antiviral [5,6] activities. In our continuing investigation of bioactive natural products obtained from Formosan soft corals, three new 9,11-secosterols, pinnisterols A–C (**1**–**3**), were obtained from a gorgonial coral identified as *Pinnigorgia* sp. (phylum Cinidaria, class Anthozoa, subclass Octocorallia, order Alcyonacea, family Gorgoniidae) (Figure 1). The structures of secosterols **1**–**3** were elucidated by spectroscopic methods and by comparison of their NMR features with those of related secosterol analogues. We report herein the isolation, structure determination and bioactivity of secosterols **1**–**3**.

## 2. Results and Discussion

The new metabolite pinnisterol A (**1**) was isolated as a colorless oil, and its molecular formula was established as C_30_H_48_O_6_ (seven degrees of unsaturation) from a sodium adduct at *m*/*z* 527 in the electrospray ionization mass spectrum (ESIMS) and further supported by a high-resolution electrospray ionization mass spectrum (HRESIMS) at *m*/*z* 527.33440 (calcd. for C_30_H_48_O_6_ + Na, 527.33431). The ^13^C and distortionless enhancement polarization transfer (DEPT) spectroscopic data of **1** showed that this compound has 30 carbons (Table 1), including seven methyls, seven sp^3^ methylenes (including an oxymethylene), seven sp^3^ methines (including two oxymethines), three sp^3^ quaternary carbons (including one oxygenated quaternary carbon), three sp^2^ methines and three sp^2^ quaternary carbons (including one ketonic carbonyl and one ester carbonyl). The IR spectrum of **1** revealed the presence of hydroxy (ν_max_ 3546 cm^−1^), ester (ν_max_ 1736 cm^−1^) and α,β-unsaturated ketone (ν_max_ 1683 cm^−1^) groups. The latter structural feature was confirmed by the presence of signals at δ_C_ 204.9 (C-9), 139.5 (CH-7) and 136.6 (C-8) in the ^13^C NMR spectrum. A disubstituted olefin was identified from the signals of carbons at δ_C_ 134.3 (CH-22) and 133.1 (CH-23), and was confirmed by two olefin proton signals at δ_H_ 5.24 (1H, m, H-22) and 5.22 (1H, m, H-23) (Table 1). Four doublets at δ_H_ 1.04 (3H, *J* = 6.8 Hz), 0.81 (3H, *J* = 6.8 Hz), 0.83 (3H, *J* = 7.2 Hz) and 0.91 (3H, *J* = 6.8 Hz) were due to the H_3_-21, H_3_-27, H_3_-26 and H_3_-28 methyl groups, respectively. Two sharp singlets for H_3_-18 and H_3_-19 appeared at δ_H_ 0.74 and 1.31, respectively. In the ^1^H NMR spectrum, one acetyl methyl signal (δ_H_ 2.00, 3H, s) was observed. Therefore, metabolite **1** must be a tricyclic compound.

**Table 1:** ^1^H (400 MHz, CDCl_3_) and ^13^C (100 MHz, CDCl_3_) NMR data and ^1^H–^1^H COSY and HMBC correlations for secosterol **1**.

Position	δ_H_ (*J* in Hz)	δ_C_, Multiple	^1^H–^1^H COSY	HMBC
1	1.94 m; 1.60 m	27.6, CH_2_	H_2_-2	C-5
2	1.91 m; 1.48 m	30.2, CH_2_	H_2_-1, H-3	n. o. ^b^
3	3.99 br s ^a^	67.1, CH	H_2_-2, H_2_-4	C-5
4	2.07 dd (12.0, 12.0); 1.74 br d (12.0)	38.7, CH_2_	H-3	C-2, -3, -5
5		76.7, C		
6	4.00 br s ^a^	71.7, CH	H-7	C-5, -7, -8, -10
7	6.40 d (4.4)	139.5, CH	H-6	C-5, -8, -9, -14
8		136.6, C		
9		204.9, C		
10		48.0, C		
11	4.14 t (7.2)	61.6, CH_2_	H_2_-12	C-12, -13, acetate carbonyl
12	1.65 m; 1.25 m	36.5, CH_2_	H_2_-11	C-11, -13, -14, -17, -18
13		45.9, C		
14	3.19 dd (9.6, 9.2)	42.6, CH	H_2_-15	C-7, -8, -9, -12, -13, -15, -18
15	1.60 m	26.9, CH_2_	H-14, H_2_-16	n. o.
16	1.69 m; 1.47 m	25.5, CH_2_	H_2_-15, H-17	C-15
17	1.69 m	50.5, CH	H_2_-16, H-20	C-15, -16
18	0.74 s	17.2, CH_3_		C-12, -13, -14, -17
19	1.31 s	21.7, CH_3_		C-1, -5, -9, -10
20	2.19 m	38.7, CH	H-17, H_3_-21, H-22	C-16, -17, -22, -23
21	1.04 d (6.8)	21.6, CH_3_	H-20	C-17, -20, -22
22	5.25 dd (15.2, 6.4)	134.3, CH	H-20, H-23	C-20, -23, -24
23	5.21 dd (15.2, 6.8)	133.1, CH	H-22, H-24	C-20, -22, -24, 28
24	1.85 q (6.4)	43.1, CH	H-23, H-25, H_3_-28	C-22, -23, -25, -26, -27, -28
25	1.45 m	33.1, CH	H-24, H_3_-26, H_3_-27	C-23, -24, -26, -27, -28
26	0.83 d (7.2)	20.0, CH_3_	H-25	C-24, -25, -27
27	0.81 d (6.8)	19.7, CH_3_	H-25	C-24, -25, -26
28	0.91 d (6.8)	17.6, CH_3_	H-24	C-23, -24, -25
11-OAc		171.7, C		
	2.00 s	21.2, CH_3_		Acetate carbonyl

^a^ Signals overlapped; ^b^ n. o. = not observed.

**Figure 2:**
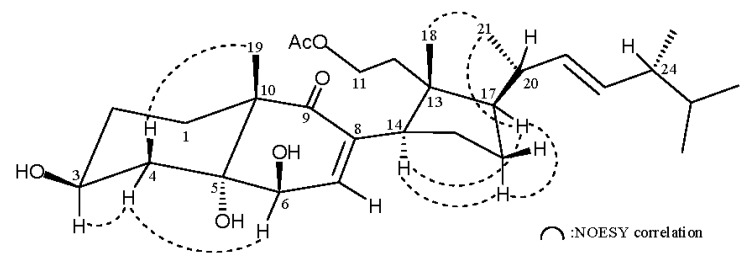
Selected NOESY correlations observed for **1**.

**Figure 3:**
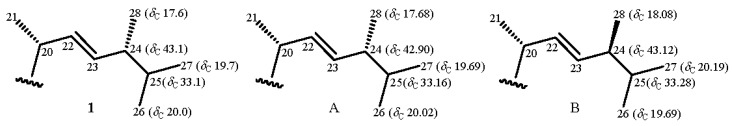
The ^13^C NMR chemical shifts of the side-chain of pinnisterol A (**1**), (22*E*,24*R*)-24-methyl- cholesta-5,22-dien-3β-ol (**A**) and (22*E*,24*S*)-24-methylcholesta-5,22-dien-3β-ol (**B**) [8].

^1^H NMR coupling information in the ^1^H–^1^H correlation spectroscopy (COSY) spectrum of **1** enabled identification of H_2_-1/H_2_-2/H-3/H_2_-4, H-6/H-7, H_2_-11/H_2_-12, H-14/H_2_-15/H_2_-16/H-17/H-20/ H-22/H-23/H-24/H-25/H_3_-26(H_3_-27), H-20/H_3_-21 and H-24/H_3_-28 (Table 1). These data, together with the key HMBC correlations between protons and quaternary carbons, such as H_2_-1, H-3, H_2_-4, H-6, H-7, H_3_-19/C-5; H-6, H-7, H-14/C-8; H-7, H-14, H_3_-19/C-9; H-6, H_3_-19/C-10; and H_2_-11, H_2_-12, H-14, H_3_-18/C-13, permitted the elucidation of the main carbon skeleton of **1** (Table 1). The relative configuration of **1** was elucidated from the correlations observed in a nuclear Overhauser effect spectroscopy (NOESY) experiment and by comparison of NMR data with those of a known secosterol, aplidiasterol B (3β,5α,6β,11-tetrahydroxy-9,11-secocholest-7-en-9-one) (**4**) (Figure 1), isolated from a Mediterranean ascidian *Aplidium conicum* [7]. The relative stereochemistries at C-3, C-5, C-6, C-10, C-13, C-14 and C-17 in **1** were found to be the same as those of **4**. Key NOE correlations for **1** showed interactions between H-3/H-4α (δ_H_ 1.74) and H-4α/H-6. Thus, H-3 and H-6 should be positioned on the α-face (Figure 2). A large coupling constant observed between H-22 and H-23 (*J* = 15.2 Hz) supported a *trans* relationship between H-22 and H-23. A stereogenic center (C-24) was identified in the side chain. The configuration at C-24 was suggested to be R* on the basis of the ^13^C NMR chemical shift of C-28 (δ_C_ 17.6). It was reported that the ^13^C NMR value of C-28 resonates at δ_C_ 17.68 ppm in the 24R* epimer of a known sterol, (22*E*,24*R*)-24-methylcholesta-5,22-dien-3β-ol, with the same chain, and the 24S* epimer, (22*E*,24*S*)-24-methylcholesta-5,22-dien-3β-ol, has a relative 0.4 ppm downfield chemical shift (Figure 3) [8]. Based on the above findings, the structure, including the relative configuration of **1**, was suggested.

Pinnisterol B (**2**) was isolated as a colorless oil, and its molecular formula was established as C_32_H_52_O_8_ (seven degrees of unsaturation) by HRESIMS at *m*/*z* 587.35558 (calcd. for C_32_H_52_O_8_ + Na, 587.35544). The IR spectrum of **2** indicated the presence of hydroxy (3420 cm^−1^), ester (1728 cm^−1^) and α,β-unsaturated ketone (1678 cm^−1^) groups. The whole series of spectroscopic data obtained from one-dimensional (1D) and two-dimensional (2D) NMR experiments (Table 2) clearly indicated that secosterol **2** had the same core structure as secosterol **1**, the differences being limited to the presence in **2** of the addition of an acetoxy group to substitute the alkene at C-23. The ^1^H and ^13^C NMR data assignments of pinnisterol B (**2**) were compared with the values of **1**. The HMBC correlations observed fully supported the locations of the functional groups, and, hence, pinnisterol B (**2**) was assigned as structure **2**, with the same relative configurations as secosterol **1** in the core rings A–C; the chiral carbons C-3, C-5, C-6, C-10, C-13, C-14 and C-17 of **2** were identical to those of **1**, and the ^1^H and ^13^C NMR chemical shifts and proton coupling constants were also in agreement.

**Table 2:** ^1^H (400 MHz, CDCl_3_) and ^13^C (100 MHz, CDCl_3_) NMR data and ^1^H–^1^H COSY and HMBC correlations for secosterol **2**.

Position	δ_H_ (*J* in Hz)	δ_C_, Multiple	^1^H–^1^H COSY	HMBC
1	1.99 m; 1.70 m	27.6, CH_2_	H_2_-2	C-19
2	1.99 m; 1.55 m	30.5, CH_2_	H_2_-1, H-3	n. o. ^a^
3	4.07 m	67.1, CH	H_2_-2, H_2_-4	C-5
4	2.14 dd (12.4, 12.0); 1.78 dd (12.4, 3.2)	39.2, CH_2_	H-3	C-2, -3, -5, -10
5		76.8, C		
6	4.05 m	72.2, CH	H-7	C-5, -7, -8, -10
7	6.43 d (5.2)	138.2, CH	H-6	C-5, -9, -14
8		137.5, C		
9		203.2, C		
10		48.2, C		
11	4.15 m	61.2, CH_2_	H_2_-12	C-12, acetate carbonyl
12	1.67 m; 1.28 m	36.8, CH_2_	H_2_-11	C-11, -13, -14, -17
13		46.1, C		
14	3.27 dd (10.8, 9.2)	42.6, CH	H_2_-15	C-7, -8, -13, -15, -18
15	1.65 m	27.0, CH_2_	H-14, H_2_-16	C-14
16	1.87 m; 1.47 m	26.1, CH_2_	H_2_-15, H-17	C-13
17	1,66 m	51.1, CH	H_2_-16, H-20	C-13, -14, -20, -21, -22
18	0.76 s	17.1, CH_3_		C-12, -13, -14, -17, -23
19	1.36 s	21.8, CH_3_		C-1, -5, -9, -10
20	1.53 m	33.4, CH	H-17, H_3_-21, H_2_-22	C-22
21	1.02 d (6.8)	20.4, CH_3_	H-20	C-17, -20
22	1.70 m; 1.22 m	35.6, CH_2_	H-20, H-23	C-17, -20, -21, -23, -24
23	5.02 m	76.9, CH	H_2_-22, H-24	n. o.
24	1.49 m	42.7, CH	H-23, H-25, H_3_-28	C-22, -23, -25, -26, -27, -28
25	1.58 m	28.5, CH	H-24, H_3_-26, H_3_-27	C-23, -24, 26, -27, -28
26	0.94 d (6.8)	21.6, CH_3_	H-25	C-24, -25, -27
27	0.84 d (6.8)	21.5, CH_3_	H-25	C-24, -25, -26
28	0.81 d (6.8)	11.0, CH_3_	H-24	C-23, -24, -25
11-OAc		171.2, C		
	2.00 s	21.1, CH_3_		Acetate carbonyl
23-OAc		170.8, C		
	2.03 s	21.5, CH_3_		Acetate carbonyl

^a^ n. o. = not observed.

Pinnisterol C (**3**) was isolated as a colorless oil. The molecular formula of **3** was established as C_32_H_52_O_8_ (seven degrees of unsaturation) from a [M + Na]^+^ ion at *m*/*z* 629.36609 in HRESIMS (calcd. for C_32_H_52_O_8_ + Na, 629.36600). The gross structure of **3** was established by interpretation of 1D and 2D NMR data, especially by analysis of ^1^H–^1^H COSY and HMBC correlations (Table 3). It was found that the NMR signals of **3** were similar to those of **2**, except that the signals corresponding to the 3-hydroxy group in **2** were replaced by signals for an acetoxy group in **3**. The correlations obtained from a NOESY experiment of **3** also showed that the configurations of chiral centers in the core rings A–C in **3** were identical to those of **2**. However, the configurations of chiral carbons C-23 and C-24 of secosterols **2** and **3** were not determined at this stage.

**Table 3:** ^1^H (400 MHz, CDCl_3_) and ^13^C (100 MHz, CDCl_3_) NMR data and ^1^H–^1^H COSY and HMBC correlations for secosterol **3**.

Position	δ_H_ (*J* in Hz)	δ_C_, Multiple	^1^H–^1^H COSY	HMBC
1	2.06 m; 1.71 m	27.4, CH_2_	H_2_-2	C-5, -10
2	1.98 m; 1.61 m	26.4, CH_2_	H_2_-1, H-3	C-3
3	5.10 m	70.3, CH	H_2_-2, H_2_-4	n. o. ^a^
4	2.21 dd (13.2, 11.6); 1.87 m	35.5, CH_2_	H-3	C-3, -5, -10, -12
5		76.5, C		
6	4.03 d (4.8)	72.3, CH	H-7	C-5, -7, -8, -10
7	6.41 d (4.8)	138.1, CH	H-6	C-5, -9, -14
8		137.4, C		
9		202.8, C		
10		48.0, C		
11	4.16 m	61.2, CH_2_	H_2_-12	C-12, -13, acetate carbonyl
12	1.61 m; 1.28 m	36.8, CH_2_	H_2_-11	C-11, -13, -14, -17
13		46.1, C		
14	3.27 dd (11.6, 8.0)	42.6, CH	H_2_-15	C-7, -8, -13
15	1.61 m	27.0, CH_2_	H-14, H_2_-16	C-4, -13
16	1.87 m; 1.47 m	26.1, CH_2_	H_2_-15, H-17	n. o.
17	1.68 m	51.2, CH	H_2_-16, H-20	n. o.
18	0.75 d (6.4)	17.1, CH_3_		C-12, -13, -14, -17
19	1.31 s	21.6, CH_3_		C-1, -5, -10
20	1.53 m	33.4, CH	H-17, H_3_-21, H_2_-22	n. o.
21	1.03 d (6.4)	20.4, CH_3_	H-20	C-17, -20, -22
22	1.71 m; 1.26 m	35.5, CH_2_	H-20, H-23	C-21, -23
23	5.02 m	76.9, CH	H_2_-22, H-24	n. o.
24	1.49 m	42.8, CH	H-23, H-25, H_3_-28	C-22, -23, -27, -28
25	1.57 m	28.5, CH	H-24, H_3_-26, H_3_-27	C-24, -27, -28
26	0.94 d (6.4)	21.6, CH_3_	H-25	C-24, -25, -27
27	0.84 d (6.4)	18.6, CH_3_	H-25	C-24, -25, -26
28	0.81 d (6.4)	11.0, CH_3_	H-24	C-23, -24, -25
3-OAc		170.8, C		
	2.01 s	21.4, CH_3_		Acetate carbonyl
11-OAc		171.1, C		
	2.00 s	21.1, CH_3_		Acetate carbonyl
23-OAc		170.8, C		
	2.05 s	21.5, CH_3_		Acetate carbonyl

^a^ n. o. = not observed.

In the biological activity testing, secosterols **1** and **3** displayed significant inhibitory effects on the release of elastase (IC_50_ = 3.32 and 2.81 μM) and inhibitory effects on the generation of superoxide anions (IC_50_ = 2.33 and 2.50 μM) by human neutrophils (Table 4) [9,10]. Secosterol **2** did not show activity in the anti-inflammatory test, which indicated that an acetoxy substituent at C-3 would enhance the activity by comparison with the structure and anti-inflammatory data of **2** with those of **3**.

**Table 4:** Inhibitory effects of compounds **1**–**3** on elastase release and superoxide anion generation by human neutrophils in response to fMet-Leu-Phe/Cytochalastin B.

	Elastase Release	Superoxide Anion
Compound	IC_50_ (μM) ^a^	IC_50_ (μM) ^a^
**1**	3.32	2.33
**2**	>10	>10
**3**	2.81	2.50

^a^ Concentration necessary for 50% inhibition (IC_50_).

**Figure 4:**
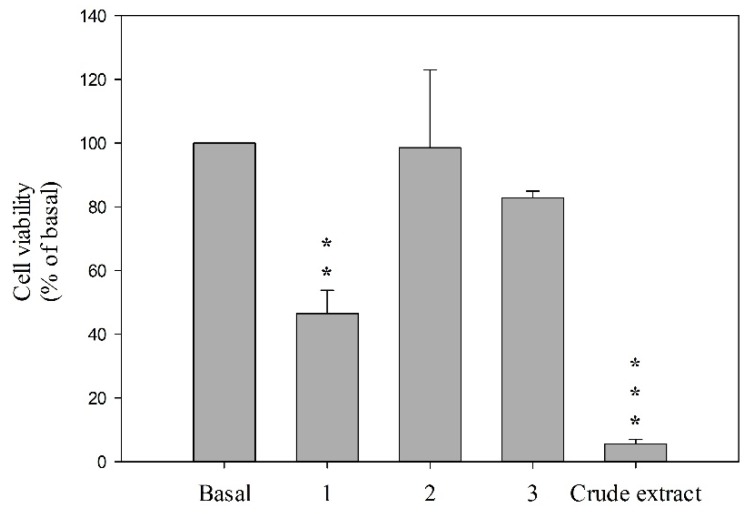
Compounds **1**–**3** decreased viability of HSC-T6 in 10 μM for 24 h. Cells were treated with DMSO (control) and coral crude extract at 6 μg/mL. Cytotoxicity assay was monitored spectrophotometrically at 450 nm. Quantitative data are expressed as the mean ± S.E.M. (*n* = 3–4). ** *p* < 0.01, *** *p* < 0.001 compared to basal.

Furthermore, in the cytotoxicity testing, secosterol **1** was found to show moderate cytotoxicity towards the HSC-T6 cells at a concentration of 10 μM (inhibition rate 46.5%) after 24 h testing and secosterols **2** and **3** were not active at the highest concentration tested (Figure 4), implying that the functional groups in the side-chain of secosterols **1**–**3** would influence the activity.

## 3. Experimental Section

### 3.1. General Experimental Procedures

Optical rotations were measured on a Jasco P-1010 digital polarimeter (Japan Spectroscopic Corporation, Tokyo, Japan). Infrared spectra were recorded on a Jasco FT/IR-4100 spectrometer (Japan Spectroscopic Corporation, Tokyo, Japan); peaks are reported in cm^−1^. The NMR spectra were recorded on a Varian Mercury Plus 400 spectrometer, using the residual CHCl_3_ signal (δ_H_ 7.26 ppm) as an internal standard for ^1^H NMR and CDCl_3_ (δ_C_ 77.1 ppm) for ^13^C NMR; coupling constants (*J*) are given in Hz. ESIMS and HRESIMS were recorded using a Bruker 7 Tesla solariX FTMS system (Bruker, Bremen, Germany). Column chromatography was performed on silica gel (230–400 mesh, Merck, Darmstadt, Germany). TLC was carried out on precoated Kieselgel 60 F_254_ (0.25 mm, Merck, Darmstade, Germany); spots were visualized by spraying with 10% H_2_SO_4_ solution followed by heating. Normal-phase HPLC (NP-HPLC) was performed using a system comprised of a Hitachi L-7110 pump (Hitachi Ltd., Tokyo, Japan) and a Rheodyne 7725 injection port (Rheodyne LLC, Rohnert Park, CA, USA). A semi-preparative normal-phase column (Supelco Ascentis Si Cat #:581515-U, 25 cm × 21.2 mm, 5 μm, Sigma-Aldrich, St. Louis, MO, USA) was used for NP-HPLC. Reversed-phase HPLC (RP-HPLC) was performed using a system comprised of a Hitachi L-2130 pump (Hitachi Ltd., Tokyo, Japan), a Hitachi L-2455 photodiode array detector (Hitachi Ltd., Tokyo, Japan), and a Rheodyne 7725 injection port (Rheodyne LLC., Rohnert Park, CA, USA). A reverse phase column (Luna^®^ 5 μm C18(2) 100Å, AXIA Packed, 25 cm × 21.2 mm, Phenomenex Inc., Torrance, CA, USA) was used for RP-HPLC.

### 3.2. Animal Material

Specimens of the gorgonian corals *Pinnigorgia* sp. were collected by hand using scuba off the coast of Green Island, Taiwan in August 2012 and stored in a freezer until extraction. A voucher specimen (NMMBA-TW-GC-2012-130) was deposited in the National Museum of Marine Biology & Aquarium, Taiwan. This organism was identified by comparison with previous descriptions [11].

### 3.3. Extraction and Separation

Sliced bodies of *Pinnigorgia* sp. (wet weight 1.98 kg; dry weight 0.86 kg) were extracted with ethyl acetate (EtOAc) at room temperature. The EtOAc extract (84.9 g) was partitioned between methanol (MeOH) and *n*-hexane. The MeOH layer (12.6 g) was separated on Sephadex LH-20 and eluted using a mixture of dichloromethane (DCM) and MeOH (1:1) to yield seven subfractions A–F. Fraction F was separated by silica gel column chromatography and eluted using *n*-hexane/acetone (stepwise, 1:1–pure acetone) to afford eight subfractions F1–F8. Fraction F2 was purified by silica gel column chromatography and eluted using *n*-hexane/acetone (stepwise, 9:1–pure acetone) to yield 13 subfractions F2A–F2M. Fraction F2G was purified by NP-HPLC using a mixture of *n*-hexane/EtOAc (1:1) to afford **3** (2.4 mg). Fraction F4 was purified by NP-HPLC using a mixture of *n*-hexane/acetone (2:1) to yield nine subfractions F4A–F4I. Fraction F4I was repurified by NP-HPLC using *n*-hexane/acetone (2:1) to afford nine subfractions F4I1–F4I9. Fraction F4I6 was separated by NP-HPLC using a mixture of *n*-hexane and acetone (2:1) to afford five subfractions F4I6A–F4I6E. Fraction F4I6C was purified by RP-HPLC, using a mixture of MeOH/H_2_O (9:1) to yield **1** (16.3 mg). Fraction F4I8 was purified by RP-HPLC using MeOH/H_2_O (85:15) to afford **2** (3.4 mg).

Pinnisterol A (**1**): colorless oil; ${\left[\alpha \right]}_{D}^{25}$ −41 (*c* 0.82, CHCl_3_); IR (neat) ν_max_ 3546, 1736, 1683 cm^−1^; ^1^H and ^13^C NMR data, see Table 1; ESIMS *m*/*z* 527 [M + Na]^+^; HRESIMS *m*/*z* 527.33440 (calcd. for C_30_H_48_O_6_ + Na, 527.33431).

Pinnisterol B (**2**): colorless oil; ${\left[\alpha \right]}_{D}^{25}$ −31 (*c* 1.13, CHCl_3_); IR (neat) ν_max_ 3420, 1728, 1678 cm^−1^; ^1^H and ^13^C NMR data, see Table 2; ESIMS *m*/*z* 587 [M + Na]^+^; HRESIMS *m*/*z* 587.35558 (calcd. for C_32_H_52_O_8_ + Na, 587.35544).

Pinnisterol C (**3**): colorless oil; ${\left[\alpha \right]}_{D}^{25}$ −39 (*c* 0.12, CHCl_3_); IR (neat) ν_max_ 3453, 1731, 1682 cm^−1^; ^1^H and ^13^C NMR data, see Table 3; ESIMS *m*/*z* 629 [M + Na]^+^; HRESIMS *m*/*z* 629.36609 (calcd. for C_34_H_54_O_9_ + Na, 629.36600).

### 3.4. Anti-Hepatofibric Assay

The anti-hepatofibric effects of tested secosterols **1**–**3** were assayed using a WST-1 assay method. Anti-hepatofibric assays were carried out according to the procedures described previously [12].

### 3.5. Generation of Superoxide Anions and Release of Elastase by Human Neutrophils

Human neutrophils were obtained by means of dextran sedimentation and Ficoll centrifugation. Measurements of superoxide anion generation and elastase release were carried out according to previously described procedures [9,10]. Briefly, superoxide anion production was assayed by monitoring the superoxide dismutase-inhibitable reduction of ferricytochrome *c*. Elastase release experiments were performed using MeO-Suc-Ala-Ala-Pro-Valp-nitroanilide as the elastase substrate.

## 4. Conclusions

Our continuing investigations demonstrated that octocorals belonging to the genus *Pinnigorgia* are good sources of 9,11-secosterols. Pinnisterols A (**1**) and B (**2**) are potentially anti-inflammatory, and may become lead compounds in future marine anti-inflammation drug development [13,14]. The results of this study suggested that continuing investigation of new secosterols together with examination of the potentially useful bioactivities of this marine organism is worthwhile for future drug development.
